# Detection of BRAF Mutation in Urine DNA as a Molecular Diagnostic for Canine Urothelial and Prostatic Carcinoma

**DOI:** 10.1371/journal.pone.0144170

**Published:** 2015-12-09

**Authors:** Hiroyuki Mochizuki, Susan G. Shapiro, Matthew Breen

**Affiliations:** 1 Department of Molecular Biomedical Sciences, College of Veterinary Medicine, North Carolina State University, Raleigh, North Carolina, United States of America; 2 Comparative Medicine Institute, North Carolina State University, Raleigh, North Carolina, United States of America; 3 Center for Human Health and the Environment, North Carolina State University, Raleigh, North Carolina, United States of America; 4 Lineberger Comprehensive Cancer Center, University of North Carolina, Chapel Hill, North Carolina, United States of America; University of Verona, ITALY

## Abstract

Urothelial carcinoma (UC) of the lower urinary tract and prostatic carcinoma (PC) are aggressive genitourinary cancers in dogs, characterized by invasion to surrounding tissues and high metastatic potential. Current diagnosis of canine UC and PC requires histopathological examination of a biopsy. Such specimens require specialized medical equipment and are invasive procedures, limiting the availability of diagnosis by histopathology for many canine patients. Access to a non-invasive means to confirm diagnosis is currently an unmet need. Recently, the canine *BRAF* V595E mutation was detected in ~80% of canine UCs and PCs. In this study, we developed a droplet digital PCR (ddPCR) assay for detection of the canine *BRAF* V595E mutation in canine urogenital tumors. The assay was evaluated in DNA samples prepared from biopsy specimens of UC (n = 48) and PC (n = 27), as well and non-neoplastic bladder epithelium (n = 38). In addition the assay was assessed for use with DNA isolated from free catch urine samples derived from canine patients with UC (n = 23), PC (n = 3), as well as from dogs with cystitis and healthy controls (n = 37). In all cases the sensitivity to detect the mutant allele was compared with conventional Sanger sequencing. ddPCR had superior sensitivity for detection of the V595E mutation: 75% of UC, 85% of PC, and 0% of control samples were mutation positive, respectively, and the V595E mutation was detected at a level as low as just 1 in 10,000 alleles (~0.01%). Furthermore, the ddPCR assay identified the mutation in free catch urine samples from 83% of canine UC and PC patients, demonstrating its utility as a non-invasive means of diagnosis. We have shown that ddPCR is a sensitive molecular technique with the potential to facilitate accurate and non-invasive means of canine UC and PC diagnosis.

## Introduction

Urothelial carcinoma (UC) of the lower urinary tract and prostatic carcinoma (PC) in dogs are characterized by local invasion and high rates of regional and distant metastases. The aggressiveness of these tumors is thought, at least in part, to result from delayed diagnosis and therapeutic intervention. Clinical symptoms of these genitourinary cancers, including hematuria, stranguria and incontinence, are indistinguishable from other non-neoplastic and more common conditions such as cystitis and prostatitis [1–3]. Currently, reliable tests to distinguish UC and PC from differential diagnoses are limited to histopathologic evaluation, requiring an invasive and costly biopsy. In addition, the size and sex of affected dogs may limit the viability of such diagnostics in individual patients, while the necessary skills and equipment may not be available in all veterinary clinics [4]. Prompt diagnosis of genitourinary cancers therefore presents a clinical challenge to veterinarians, potentially impeding the timeline of appropriate therapy.

Less invasive diagnostic aides for canine genitourinary cancers are available, though these are considered unreliable. The presence of abnormal epithelial cells in urine sediment, traumatic catheterization, prostatic wash and/or fine needle aspiration have all been used to support the diagnosis of canine UC and PC [3–5]. Cytological analysis of epithelial cells, however, may be misleading. For example, benign epithelial cells can appear morphologically neoplastic, with variation in cell size and increased basophilia; after prolonged contact with urine or under urothelial hyperplasia secondary to inflammatory condition [6]. Fine needle aspiration of tumor tissue carries the risk of disseminating tumor cells along the needle tract and should be performed with caution [7,8]. Currently, clinical diagnosis of canine UC and PC requires comprehensive diagnostic workups, including blood test, urinalysis and diagnostic imaging, in addition to cytological examinations of tumor cells by skilled clinical pathologists [4,9]. The availability of a reliable, non-invasive diagnostic test for canine UC and PC remains a paramount need.

Recent studies identified a somatic mutation in the canine *BRAF* (*cBRAF*) gene in several canine cancers, including a large proportion of canine UC and PC [10,11]. The mutation was a single nonsynonymous substitution of T to A at nucleotide 1784, resulting in the amino acid substitution from valine to glutamic acid at codon 595 (V595E, based on *cBRAF* reference sequence: Ensemble Transcript ID: ENSCAFT00000006306). Among various types of cancers of epithelial, messenchymal, hematopoitiec and other cancers of miscellaneous origin, the studies identified the V595E mutation in canine UC and PC with the highest penetrance rates of up to 87%. Since UC and PC tumor cells often exfoliate and shed into urine, the presence of the V595E mutation in urine can be a molecular diagnostic marker [12].

Despite the high prevalence of the *cBRAF* mutation, there are technical challenges in detecting the mutation in urine samples of canine UC and PC patients. Secondary bacterial cystitis is common in dogs with UC and PC, recruiting a large number of inflammatory cells and reactive epithelial cells and resulting in dilution of the shed tumor cell population in urine [4,9]. Sanger sequencing, the gold standard for detecting a single nucleotide substitution, requires a 10–20% fraction of mutated allele for reliable detection [13]. This low sensitivity leads to false negative results in a mixed cell population, such as urine samples with low neoplastic cellularity.

Digital PCR is a highly sensitive molecular technique enabling detection of a “rare” mutated sequence in clinical samples such as tumor DNA in plasma cell-free DNA, or, in this case, urine [14–16]. Digital PCR is performed by partitioning the PCR mixtures into a large number of compartments (e.g. droplets), where each compartment contains either ≥ 1 (positive) or 0 (negative) target sequences. After conventional thermal cycling amplification, each compartment is classified as being either positive or negative by assessment of the fluorescence signal intensity at the end point. Wild type and mutant alleles are detected in the same reactions, using spectrally resolvable fluorochromes. The numbers of positive and negative compartments for each fluorochrome, together with Poisson’s distribution, are used to calculate absolute number of target sequence in a sample without the need for calibration standards.

In this study, we developed a ddPCR-based *cBRAF* V595E mutation detection assay. With the ddPCR assay, we examined the presence of the V595E mutation in tissue and urine samples from dogs diagnosed with UC, PC, and in nonneoplastic control samples.

## Materials and Methods

### Tissue samples

All tissue and urine specimens used in this study were obtained by the North Carolina State University (NCSU) Histology Lab or Clinical Studies Core, following NCSU IACUC approved protocol No. 13-022-O, which included written informed consent from owners approving use in research studies. Genomic DNA (gDNA) from 48 UC (n = 48), 27 PC (n = 27), and control bladder tissues (n = 30 as normal n = 26, cystitis n = 3 and urothelial hyperplasia n = 1), and control prostate gland (all normal, n = 8) was included in the present study. These gDNA sample were derived from the archive of our previous study [11], supplemented with five additional (3 UC and 2 PC) DNA samples derived from additional formalin-fixed paraffin-embedded (FFPE) blocks of tumor tissues obtained through North Carolina State University (NCSU) Histology Lab. gDNA was isolated from FFPE blocks using a QIAamp FFPE DNA extraction kit (Qiagen, Valencia, CA). Spectrophotometry (NanoDrop, Thermo Scientific, Wilmington, DE) and agarose gel electrophoresis were used to determine DNA quantity and integrity.

### Urine samples

Free catch urine samples were collected by the NCSU Clinical Studies Core from canine patients with informed owner consent. Dogs presenting to the NCSU CVM Veterinary Teaching Hospital with one of four clinical presentations were included: 1) urothelial carcinoma of bladder and/or urethra (n = 23), 2) prostate carcinoma (n = 3), 3) bacterial cystitis (n = 10), and 4) clinically-healthy dogs (n = 27) were included. Of UC and PC patients, seven dogs were diagnosed through tumor histopathology, while 19 were diagnosed based on the presence of a mass in the trigonal region of the bladder or in the prostate gland, together with and cytological evaluation of urine sediments and/or fine needle aspirations of the mass. Genomic DNA was isolated from urine sediment after the cells were washed with PBS (14 dogs) or fixed in 3:1 methanol acetic acid fixative used for chromosomal analysis (49 dogs), some of which were used in the previous study [17]. Signalment of dogs for urine samples and DNA source is shown in S1 Table.

### Sanger sequencing analysis

The *cBRAF* V595E mutation status of all samples included in the study was evaluated by Sanger sequencing, combined with data obtained in the past study [11]. PCR amplification was performed to amplify a 391-bp DNA fragment spanning the region of *cBRAF* V595E as previously described [11]. Sequencing was performed at the North Carolina State University Genome Research Laboratory (http://research.ncsu.edu/gsl/) and data analyzed for the presence of cBRAF V595E mutation using 4peaks software (http://nucleobytes.com/index.php/4peaks).

### Digital droplet PCR analysis for cBRAF V595E mutation

A ddPCR assay, comprising forward and reverse primers and TaqMan® MGB probes for the detection of wild-type and V595E mutated sequences, was designed with the aid of Primer Express Software (version 3.0.1, Life Technologies, Carlsbad, CA). Sequences and locations of primers and probes are shown in Table 1.

**Table 1:** Primers and probes used in the canine BRAF V595E ddPCR assay.

	Sequence and modification	Location on CFA 16
Forward primer	5'–TCATGAAGACCTCACAGTAAAAATAGGT–3'	8,296,237–8,296,264
Reverse primer	5'–TGGGACCCACTCCATCGA–3'	8,296,291–8,296,308
V595E Mut probe	5'–FAM–TCTAGCCACAGAGAAA–MGB–3'	8,296,273–8,296,288
Wt probe	5'–VIC–TAGCCACAGTGAAAT–MGB–3'	8,296,275–8,296,289

CFA, canine chromosome; Mut, mutant allele; Wt, wild-type allele

The cBRAF V595E ddPCR assay comprised 20 μL of 1 x Droplet Supermix (Bio-Rad Laboratories, Richmond, CA), ~20 ng of genomic DNA isolated from each patient sample, 2 U of *Mse*I restriction enzyme (New England Biolabs, Ipswich, MA), 500 nM of forward and reverse primers and 250 nM of VIC- and FAM-labeled TaqMan® MGB probes for wild-type and the V595E sequences, respectively. The PCR reaction mixtures were partitioned into an emulsion of ~20,000 droplets (number of droplets measured: mean ± SD: 14,648 ± 2,127) using a QX200™ ddPCR system (both from Bio-Rad). PCR was performed on T100™ Thermal Cycler using thermal cycle condition as follows: denaturation at 95°C for 10 min; 40 cycles of 94°C for 30 sec and 59°C for 60 sec; 98°C for 10 min. Post PCR, droplets were analyzed on QX200™Droplet Reader (Bio-Rad). The concentrations of wild-type and V595E sequences were calculated on the Poisson distribution using the Quantasoft™ software Version 1.7.4 (Bio-Rad). Wild-type control DNA (prepared from a histopathologically normal lymph node) and non-template control reactions were included in each experiment. Up to three positive droplets for the V595E assay were occasionally seen in the wild-type control samples in the preliminary setting (false-positive). In this study only reactions containing at least five single occupancy V595E-positive droplets were classified as positive in the ddPCR analysis.

To determine the detection limit of mutation fraction, serial dilutions of a plasmid containing BRAF V595E mutation were prepared. To prepare the plasmid containing V595E sequence, PCR products obtained from a BRAF positive canine prostate carcinoma cell line, Leo (kindly provided from Dr. T. Rosol, [18]), were cloned using a pGEM®-T Easy Vector Systems (Promega, Madison, WI). Plasmid DNA containing the V595E sequence was used to make a dilution series in 200 ng of wild type genomic dog DNA, where the mutation fractions were 50%, 10%, 5%, 1%, 0.5%, 0.1%, 0.05%, 0.01%, 0.005% and 0.001%. Since 20 ng of DNA may not be available in a clinical setting, to assess the effects of DNA amount in the ddPCR reaction, a DNA dilution series of four urine DNA specimens from UC patients (1, 2, 5, 10, 20, 50 and 200 ng/reaction) was also prepared and analyzed by ddPCR.

## Results

**Fig 1:**
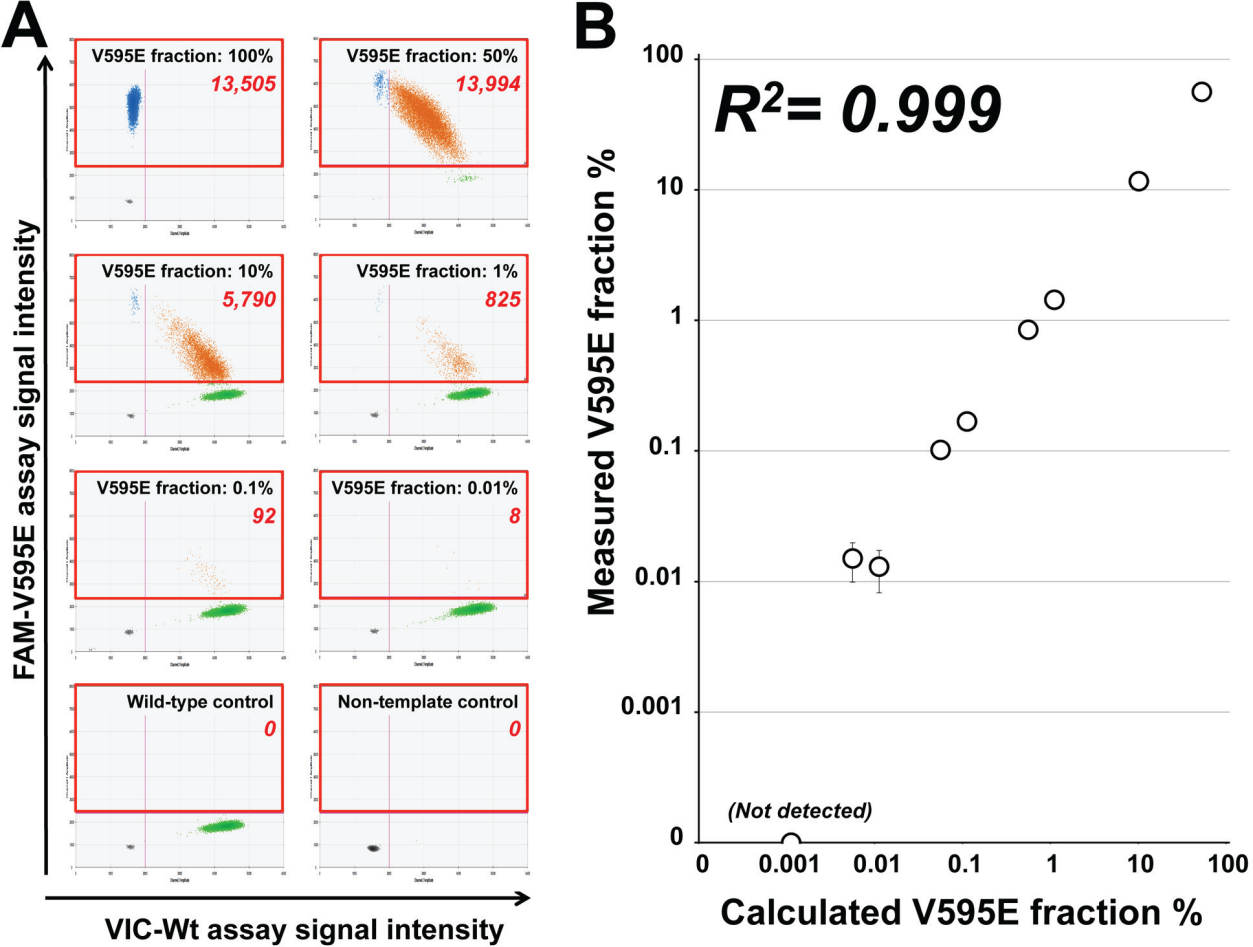
Performance of the cBRAF V595E assay in a serial dilution of plasmid containing V595E sequence in wild type dog gDNA. (A) Representative two-dimensional scatter plots of the ddPCR assay in serially-diluted DNA samples. Areas in red rectangles indicate the positive reactions for cBRAF V595E assay. The numbers shown in red rectangles indicate the number of droplets positive for the V595E mutation assay. (B) Correlation of V595E fraction calculated by the dilutions and measured by ddPCR. The V595E mutation was detected up to 0.005% of V595E fraction. Vertical error bars represents the 95% confidence intervals calculated by the Poisson distribution.

We designed a ddPCR assay for the detection of the *cBRA*F V595E mutation. To investigate the performance of the *cBRAF* V595E ddPCR assay, plasmid DNA containing V595E sequence was serially diluted into wild type dog genomic DNA (range of 50% to 0.001%). The assay was able to detect the mutation when present at levels as low as 0.005% and showed a clear linearity between calculated and measured V595E fractions (R^2^ = 0.999, Fig 1).

**Fig 2:**
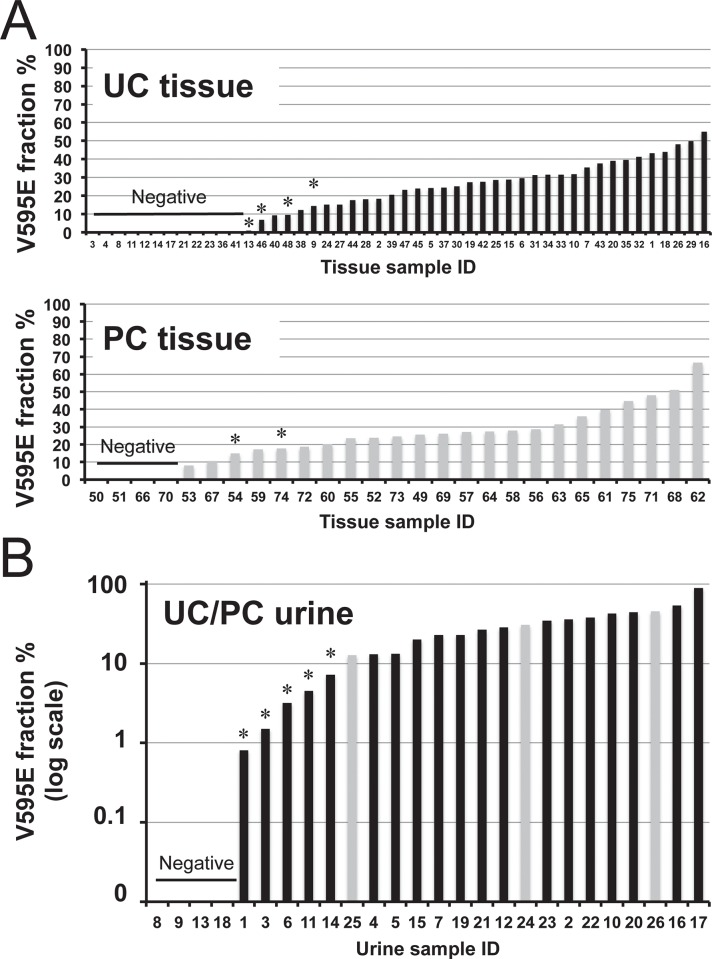
Results of *cBRAF* V595E ddPCR analysis of (A) tissue and (B) urine samples of canine UC and PC patients (x-axis: each sample, y-axis: V595E fraction %). Black and gray bars indicate UC and PC samples, respectively. Asterisks indicate samples in which the V595E mutation was not detected in Sanger sequencing analysis.

Next, we compared ddPCR and Sanger sequencing results to evaluate *cBRAF* V595E detection sensitivity in tissues samples. With Sanger sequencing we were able to detect the V595E mutation in 71% (53/75) of cases; 67% (32/48) of UC and 78% (21/27) of PC. Our ddPCR assay detected all of the cases (53/75) identified by Sanger sequencing and was able to detect the mutation in additional four UC and two PC samples, increasing the frequency of the V595E mutation overall to 79% (59/75), as 75% (36/48) and 85% (23/27) cases of canine UC and PC, respectively. The detection limit of Sanger sequencing was 10–20% of the mutation fraction (Fig 2A). Neither Sanger sequencing nor ddPCR analysis detected the mutation in any of the control bladder or prostate gland tissue samples.

We then analyzed urine DNA samples from dogs with UC and PC, as well as those with cystitis (differential diagnosis) and clinically healthy dogs. With Sanger sequencing of urine from UC patients, all but one urine-derived DNA sample yielded PCR products for sequencing analysis. Sequencing analysis revealed the V595E mutation in urine DNA samples of 61% (14/23) of UC patients and 100% (3/3) of PC patients. No control urine sample (27 healthy or 10 cystitis) was positive for the mutation. With ddPCR we detected the mutation in 83% (19/23) of UC and 100% (3/3) of PC patients, including five UC samples in which the mutation was not detected by Sanger sequencing (Fig 2B). With ddPCR the V595E mutation was not detected in any of the healthy or cystitis dog urine samples. For the diagnosis of canine UC and PC, the overall sensitivity and specificity values of the *cBRAF* V595E ddPCR assay in urine DNA were 85% (22/26) and 100% (37/37), respectively. The detection rates of the *cBRAF* V595E mutation in tissue and urine samples according to Sanger sequencing and ddPCR analysis are summarized in Table 2.

**Table 2:** Sequencing and ddPCR analysis for V595E mutation in tissue and urine.

Specimen	Disease	N	BRAF V595E mutation status			
			Sanger sequencing		ddPCR	
			+	–	+	–
Tissue	UC	48	32 (67%)	16	36 (75%)	12
	PC	27	21 (78%)	6	23 (85%)	4
	Control	38	0	38	0	38
Urine	UC	23	14 (61%)	9	19 (83%)	4
	PC	3	3 (100%)	0	3 (100%)	0
	Control	37	0	37	0	37

UC, urothelial carcinoma; PC, prostatic carcinoma

**Fig 3:**
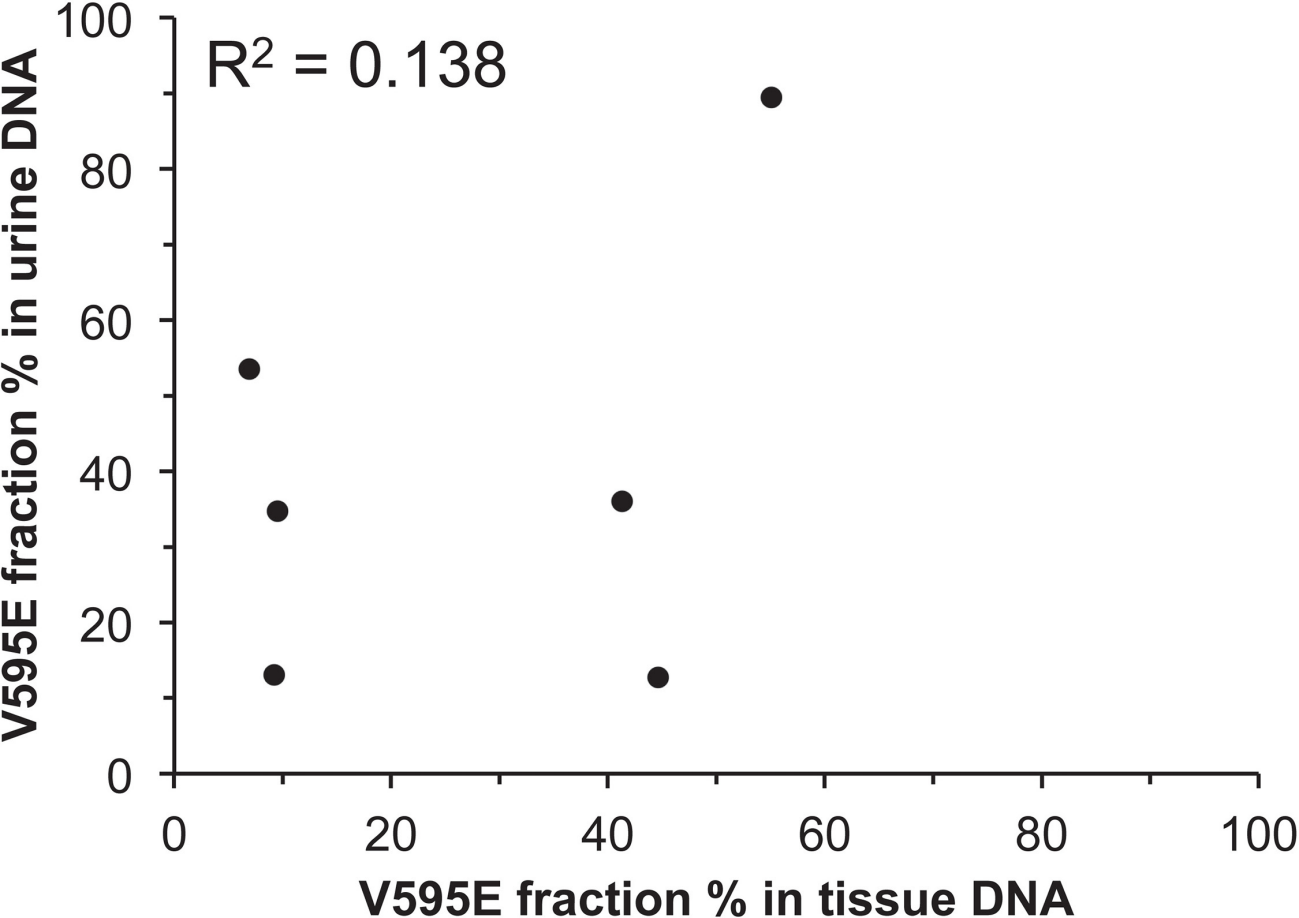
Comparison of the *cBRAF* V595E fraction between matched tissue and urine samples of six UC/PC cases. The association between tissue and urine was not evident.

To evaluate the relationship between the V595E mutation fraction obtained by ddPCR of DNA obtained from tissue and urine samples, the mutation fraction was compared between six matched tissue and urine samples. The V595E mutation was detected in both tissue and urine specimens from the six dogs by Sanger sequencing and ddPCR. As expected, there was no or little correlation between urine and tissue mutation fractions examined by ddPCR (Pearson correlation coefficient, R^2^ = 0.138, Fig 3), reflecting the variable dilution of the mutated allele with wild-type alleles derived from non-neoplastic (wild type) cells shed into the urine.

**Fig 4:**
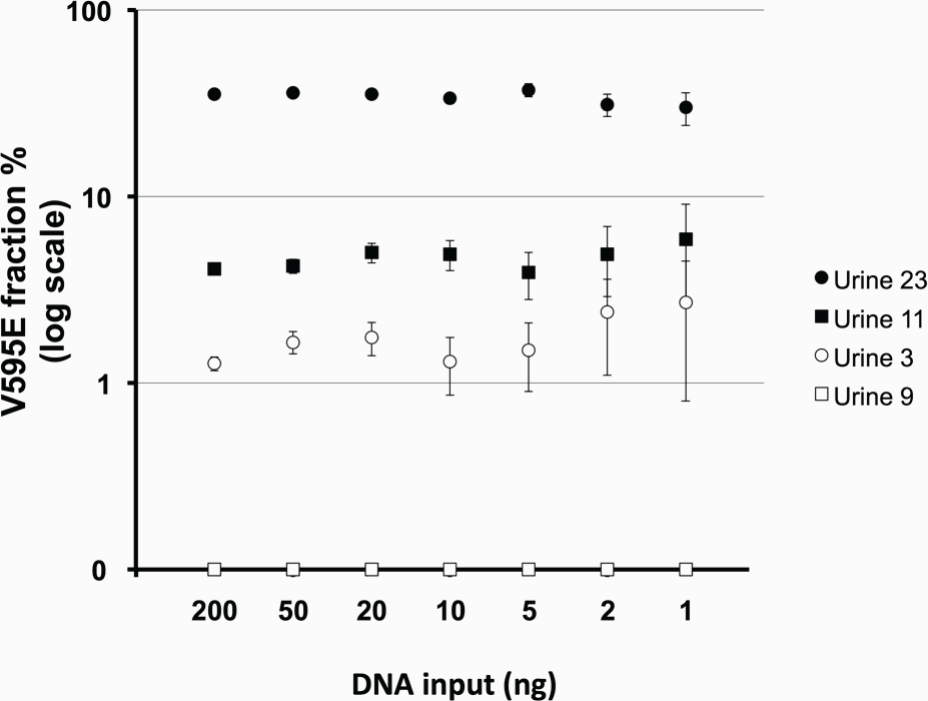
Performance of the *cBRAF* V595E ddPCR assay at 1–200 ng DNA input. The V595E fraction showed little variance between a wide range of DNA input. Vertical error bars represents the 95% confidence intervals calculated by the Poisson distribution.

Since the amount of DNA obtained from urine is generally limited in a clinical setting, we tested the performance of the ddPCR assay at a wide range of DNA input (1ng–200 ng DNA/reaction). For this we used four urine samples with V595E mutation fractions of 0% (negative), 1.5%, 4.5% and 33%. All three V595E-positive samples produced positive results with 1–200 ng of DNA input, whereas the V595E-negative samples remained negative. Regardless of different amounts of DNA input, the mutation fraction showed little variance, demonstrating that the assay was not only qualitative but also reliably quantitative at a wide range of DNA input (Fig 4).

## Discussion

A major challenge in the clinical management of canine UC and PC lies in accurate diagnosis. Due to the lack of reliable, non-invasive diagnostic tests, combined with non-specific clinical symptoms, canine UC and PC are often diagnosed at advanced stages of disease, at which time the majority (>90%) of UC are invasive and many (>20% of UC and >40% of PC) have metastasized [3,4]. Currently, histopathological evaluation of a tumor biopsy is considered the gold standard for diagnosis of these canine cancers. However, the invasive nature of tumor biopsy, risks of anesthesia, high procedure cost, and low clinical availability impede the ability for most patients to be properly diagnosed. A non-invasive, affordable, and accessible diagnostic test for these cancers is an unmet need with potential therapeutic and, therefore, prognostic impact.

Molecular detection of genetic alterations has been widely used for cancer diagnosis in human medicine [19]. Since somatic alteration of genetic components is often neoplasia-specific, their detection can provide a specific and objective marker for cancer diagnosis. The *cBRAF* V595E mutation, corresponding to human *BRAF* V600E, has recently been discovered in ~80% of canine UC and PC, rendering it of potential diagnostic significance [10,11]. In addition, BRAF inhibitors have been developed for targeted treatment of *BRAF* mutant tumors, and previous studies have shown such therapies to be highly effective in several human malignancies harboring the *BRAF* V600E mutation [20–24]. Thus, c*BRAF* V595E detection in urine may be a guide for individualized medicine in canine genitourinary tumors.

In this study, we established a sensitive ddPCR-based *cBRAF* V595E assay and evaluated its performance as a non-invasive molecular diagnostic technique for the detection of canine UC and PC. Consistent with past studies, the detection sensitivity of a mutant allele by Sanger sequencing of canine UC and PC was 10–20%. The low sensitivity of the Sanger sequencing led to false-negative results in tissue specimens (UC: 8.3%, PC: 7.4%) that was almost three times higher in urine samples (UC: 22%), underscoring the necessity of more sensitive methods to detect the V595E mutation in urine samples. Using a custom ddPCR assay, we were able to detect the V595E mutation in both tissue and urine samples of low tumor cellularity. The assay was able to detect as low as one mutant allele in the presence of 10,000 wild type alleles (0.01%) and showed high linearity in quantification of the mutated allele fraction. These data indicate that ddPCR would be a highly sensitive means of non-invasive diagnosis for canine genitourinary cancers.

Although many different molecular techniques can be used to detect somatic mutations, digital PCR is one of the most sensitive methods that can detect a point mutation when present at levels as low as 0.001–0.01% [25] In one study, digital PCR was compared to high-resolution melt curve analysis, pyrosequencing, and allele-specific amplification to detect the human *BRAF* V600E mutation in human melanoma tumor tissues [26]. Digital PCR showed superior detection limit compared to the other three methods, especially in tissue samples with mutation load of <5%. In this study, the *cBRAF* V595E mutation fraction was <5% in 21% (4/19) of mutation positive urine samples, indicating that a highly sensitive method of detection is critical for it to be clinically effective. It is of note that all canine cancer patients in this study had full-blown disease at the time of urine collection (clinically diagnosed), suggesting that the mutation load may be lower during early/subclinical stages of disease. The heterogeneity of cells shed in urine necessitates employment of a sensitive method to detect the *cBRAF* V595E mutation in canine UC and PC patients. Furthermore, the ddPCR assay provides not only qualitative results (positive vs negative for V595E) but also quantitative data of tumor-derived mutation load in urine DNA. The assay was found to be highly quantitative at wide range of mutation fractions, enabling the quantification of tumor load in the urine of each patient during clinical course. Although it is still unknown if the urine tumor load reflects the entire tumor volume, the tumor load may be a potential tool to monitor treatment response and detect minimal residual diseases after surgery.

Compared to traditional quantitative PCR (qPCR)-based methods, ddPCR is more resistant to PCR inhibitors. It is well-known that PCR inhibitors are co-purified when isolating DNA from urine, compromising PCR efficiencies at various degrees [27,28]. To calculate target concentrations, qPCR uses threshold cycles (Ct) value, which is defined as the number of cycles for the fluorescent signal to cross a certain threshold. Since Ct values are highly affected by PCR efficiencies, the presence of PCR inhibitors could lead to underestimation of target concentrations or false-negative results on qPCR-based assays, especially for targets of a small number of copies. In contrast, PCR efficiency has little impact on digital PCR, as digital PCR only uses numbers of positive and negative reactions at the PCR end-point. One study demonstrated that digital PCR was 10–100 times more tolerant of inhibitors than qPCR [29]. This feature also makes ddPCR a practical means of mutation detection in PCR inhibitor-rich urine.

One limitation of the V595E mutation as a molecular marker for canine UC and PC patients is that ~20% of tumors do not possess the mutation, limiting the sensitivity of the ddPCR assay around 80%. Combination of V595E mutation with sensitive molecular techniques, such as characteristic chromosome copy number aberrations described previously, will increase the sensitivity of the molecular analysis of urine DNA [17,30]. The complementarity of the high specificity c*BRAF* V595E ddPCR assay with sensitive techniques has the potential to provide a reliable, yet noninvasive and readily accessible diagnostic option. Furthermore, potential pharmacologic targeting of *cBRAF* V595E expands the value of the assay to enable therapeutic guidance. Further studies are warranted to characterize the genomic alteration of canine UC and PC and refine the non-invasive diagnostic method to these challenging diseases, including therapeutic applications.

## Supporting Information

S1 Table Signalment of dogs for urine samples and DNA source. (DOCX)
