# Supplementary material for: Detection of BRAF Mutation in Urine DNA as a Molecular Diagnostic for Canine Urothelial and Prostatic Carcinoma
Source: PLoS One. 2015 Dec 9;10(12):e0144170. doi: 10.1371/journal.pone.0144170 (PMC4674145; doi:10.1371/journal.pone.0144170)
Supplement: S1 Table — (DOCX) [file pone.0144170.s001.docx]

S1 Table. Signalment of dogs for urine samples.

| Urine ID | Disease | Breed | Gender | Age | DNA source |
| --- | --- | --- | --- | --- | --- |
| Urine 1 | UC | Labrador Retriever | FS | 8 y | Fixed cells |
| Urine 2 | UC | Scottish Terrier | FS | 7 y | Fixed cells |
| Urine 3 | UC | Labrador Retriever | FS | 11 y | Fixed cells |
| Urine 4 | UC | Boston Terrier | FS | 9 y | Fresh cells |
| Urine 5 | UC | Beagle | F | 12 y | Fixed cells |
| Urine 6 | UC | Beagle | FS | 10 y | Fixed cells |
| Urine 7 | UC | Staffordshire Bull Terrier | MC | 11 y | Fixed cells |
| Urine 8 | UC | Rottweiler | FS | 4 y | Fixed cells |
| Urine 9 | UC | Brussels Griffon | MC | 8 y | Fresh cells |
| Urine 10 | UC | Scottish Terrier | FS | 14 y | Fresh cells |
| Urine 11 | UC | Mixed | FS | 7 y | Fresh cells |
| Urine 12 | UC | Chihuahua | FS | 9 y | Fixed cells |
| Urine 13 | UC | English Setter | FS | 9 y | Fixed cells |
| Urine 14 | UC | Chow Chow | MC | 9 y | Fresh cells |
| Urine 15 | UC | West Highland White Terrier | MC | 12 y | Fresh cells |
| Urine 16 | UC | Labrador Retriever | FS | 11 y | Fixed cells |
| Urine 17 | UC | Keeshond | MC | 10 y | Fixed cells |
| Urine 18 | UC | Dalmatian | MC | 10 y | Fresh cells |
| Urine 19 | UC | Mixed | MC | 12 y | Fresh cells |
| Urine 20 | UC | West Highland White Terrier | MC | 11 y | Fresh cells |
| Urine 21 | UC | Chihuahua | MC | 12 y | Fixed cells |
| Urine 22 | UC | German Shepherd Dog | FS | 13 y | Fresh cells |
| Urine 23 | UC | Staffordshire Bull Terrier | FS | 11 y | Fresh cells |
| Urine 24 | PC | Gordon Setter | MC | 9 y | Fresh cells |
| Urine 25 | PC | Shetland Sheepdog | MC | 8 y | Fixed cells |
| Urine 26 | PC | Silky Terrier | M | 12 y | Fixed cells |
| Urine 27 | Cystitis | Mixed | MC | 14 y | Fixed cells |
| Urine 28 | Cystitis | Beagle | MC | 8 y | Fixed cells |
| Urine 29 | Cystitis | Soft Coated Wheaten Terrier | FS | 2 y | Fixed cells |
| Urine 30 | Cystitis | Bichon Frise | FS | 12 y | Fixed cells |
| Urine 31 | Cystitis | Greyhound | FS | 10 y | Fixed cells |
| Urine 32 | Cystitis | Anatolian Shepherd Dog | F | 4 m | Fixed cells |
| Urine 33 | Cystitis | Golden Retriever | FS | 13 y | Fixed cells |
| Urine 34 | Cystitis | Pekingese | FS | 12 y | Fixed cells |
| Urine 35 | Cystitis | Labrador Retriever | M | 12 y | Fixed cells |
| Urine 36 | Cystitis | Maltese | F | 11 y | Fixed cells |
| Urine 37 | Healthy | American Staffordshire Terrier | MC | 2 y | Fixed cells |
| Urine 38 | Healthy | Bernese Mountain Dog | MC | 5 y | Fixed cells |
| Urine 39 | Healthy | Catahoula Leopard Dog | FS | 6 y | Fixed cells |
| Urine 40 | Healthy | American Staffordshire Terrier | MC | 2 y | Fixed cells |
| Urine 41 | Healthy | Basenji | FS | 10 y | Fixed cells |
| Urine 42 | Healthy | English Bulldog | FS | 4 y | Fixed cells |
| Urine 43 | Healthy | Labrador Retriever | FS | 8 y | Fixed cells |
| Urine 44 | Healthy | Golden Retriever | FS | 10 y | Fixed cells |
| Urine 45 | Healthy | Golden Retriever | MC | 9 y | Fixed cells |
| Urine 46 | Healthy | Mixed | MC | 12 y | Fixed cells |
| Urine 47 | Healthy | French Bulldog | MC | 7 y | Fixed cells |
| Urine 48 | Healthy | Yorkshire Terrier | FS | 12 y | Fixed cells |
| Urine 49 | Healthy | Labrador Retriever | MC | 3 y | Fixed cells |
| Urine 50 | Healthy | Dachshund | MC | 4 y | Fixed cells |
| Urine 51 | Healthy | American Foxhound | MC | 6 y | Fixed cells |
| Urine 52 | Healthy | Shetland Sheepdog | FS | 4 y | Fixed cells |
| Urine 53 | Healthy | Border Collie | MC | 6 y | Fixed cells |
| Urine 54 | Healthy | Mixed | FS | 4 y | Fixed cells |
| Urine 55 | Healthy | Mixed | FS | 2 y | Fixed cells |
| Urine 56 | Healthy | Mixed | FS | 3 y | Fixed cells |
| Urine 57 | Healthy | Labrador Retriever | FS | 1 y | Fixed cells |
| Urine 58 | Healthy | German Shepherd Dog | FS | 10 m | Fixed cells |
| Urine 59 | Healthy | Border Collie | FS | 4 y | Fixed cells |
| Urine 60 | Healthy | Border Collie | FS | 3 y | Fixed cells |
| Urine 61 | Healthy | Border Collie | MC | 5 y | Fixed cells |
| Urine 62 | Healthy | Beagle | MC | 5 y | Fresh cells |
| Urine 63 | Healthy | Golden Retriever | FS | 4 y | Fresh cells |
| F: female, FS: female castrated, M: male, MC: male castrated | | | | | |
